# SAMHD1 restricts HIV-1 reverse transcription in quiescent CD4^+^ T-cells

**DOI:** 10.1186/1742-4690-9-87

**Published:** 2012-10-23

**Authors:** Benjamin Descours, Alexandra Cribier, Christine Chable-Bessia, Diana Ayinde, Gillian Rice, Yanick Crow, Ahmad Yatim, Olivier Schwartz, Nadine Laguette, Monsef Benkirane

**Affiliations:** 1 Institut de Génétique Humaine, CNRS UPR1142, Laboratoires de Virologie Moléculaire, Montpellier, France; 2 Academic Unit of Medical Genetic, University of Manchester, Manchester, UK; 3 Institut Pasteur, Virus and Immunity Unit, URA CNRS 3015, Paris, France

**Keywords:** SAMHD1, Quiescent CD4^+^ T-cell, HIV-1, Reverse transcription, Restriction

## Abstract

**Background:**

Quiescent CD4^+^ T lymphocytes are highly refractory to HIV-1 infection due to a block at reverse transcription.

**Results:**

Examination of SAMHD1 expression in peripheral blood lymphocytes shows that SAMHD1 is expressed in both CD4+ and CD8+ T cells at levels comparable to those found in myeloid cells. Treatment of CD4+ T cells with Virus-Like Particles (VLP) containing Vpx results in the loss of SAMHD1 expression that correlates with an increased permissiveness to HIV-1 infection and accumulation of reverse transcribed viral DNA without promoting transcription from the viral LTR. Importantly, CD4^+^ T-cells from patients with Aicardi-Goutières Syndrome harboring mutation in the *SAMHD1* gene display an increased susceptibility to HIV-1 infection that is not further enhanced by VLP-Vpx-treatment.

**Conclusion:**

Here, we identified SAMHD1 as the restriction factor preventing efficient viral DNA synthesis in non-cycling resting CD4^+^ T-cells. These results highlight the crucial role of SAMHD1 in mediating restriction of HIV-1 infection in quiescent CD4^+^ T-cells and could impact our understanding of HIV-1 mediated CD4^+^ T-cell depletion and establishment of the viral reservoir, two of the HIV/AIDS hallmarks.

## Background

The human immunodeficiency virus type-1 (HIV-1) primarily infects CD4^+^ T-cells. While activated lymphocytes support viral replication, non-cycling quiescent CD4^+^ T-cells allow entry of HIV-1 but fail to allow efficient and complete reverse transcription [1-6]. However, addition of deoxynucleosides to unstimulated lymphocytes cultures partially overcomes this failure [7-9], suggesting that an insufficient supply of deoxynucleotide triphosphates (dNTPs) in quiescent T-cells may, at least in part, contribute to inefficient viral DNA synthesis [8,10]. Interestingly, the Aicardi-Goutières syndrome gene product SAMHD1 was recently described as the restriction factor that blocks HIV-1 infection of non-cycling myeloid cells [11-13]. SAMHD1 is a dGTP-dependent deoxynucleotide triphosphohydrolase [14-16] that reduces the cellular pool of dNTPs in differentiated, non-cycling myeloid cells to levels below those required to support HIV-1 DNA synthesis [15,17]. However, SAMHD1’s spectrum of activity beyond cells of the myeloid lineage remains unclear.

## Results and discussion

**Figure 1:**
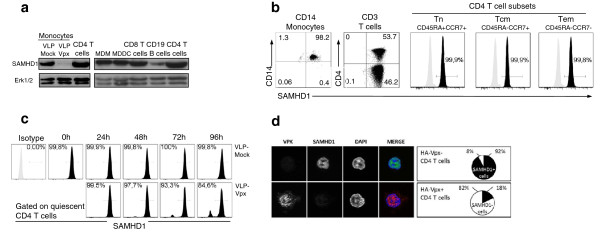
**CD4 T lymphocytes express SAMHD1.a**. Western blot analysis of SAMHD1 expression in primary hematopoietic cells. **b**. Flow cytometry analysis of SAMHD1 expression levels in CD14^+^ monocytes, T-cells (CD4^+^ and CD8^+^ T lymphocytes) and in CD4^+^ T-cell subsets including Naïve (Tn; CD45RA^+^, CCR7^+^), Central Memory (Tcm; CD45RA^-^, CCR7^+^), and Effector Memory (Tem; CD45RA^-^, CCR7^-^). **c**. Kinetics of SAMHD1 loss in quiescent (CD69^-^, HLA-DR^-^) CD4^+^ T-cells after VLP-Vpx treatment (representative experiment, n = 2). **d**. Vpx transduction of activated CD4^+^ T-cells induces loss of SAMHD1. TCR-stimulated CD4^+^ T-cells were transduced with Vpx-HA expressing retroviral construct. Forty-eight hours later, SAMHD1 and Vpx were immunostained using specific antibodies. Nuclei were stained in mounting media with DAPI. Pie charts represent the proportions of SAMHD1^+^ cells among 100 Vpx positive and negative counted cells.

We first determined whether SAMHD1 was expressed in peripheral blood lymphocytes from healthy donors. Western blot analysis revealed that both CD4^+^ and CD8^+^ T-cells express SAMHD1 (Figure 1a). The expression levels of SAMHD1 in unstimulated CD4^+^ T-cells were similar to those found in myeloid cells, including CD14^+^ monocytes, monocytes-derived macrophages (MDM) and monocyte-derived dendritic cells (MDDC) (Figure 1a). Moreover, flow cytometry analysis revealed that all circulating CD4^+^ T-cells subsets, including naïve (Tn; CD45RA^+^, CCR7^+^), central memory (Tcm; CD45RA^-^, CCR7^+^) and effector memory cells (Tem; CD45RA^-^, CCR7^-^), expressed high levels of SAMHD1 (Figure 1b), raising the possibility that SAMHD1 could affect the susceptibility of resting CD4^+^ lymphocytes to HIV-1 infection.

**Figure 2:**
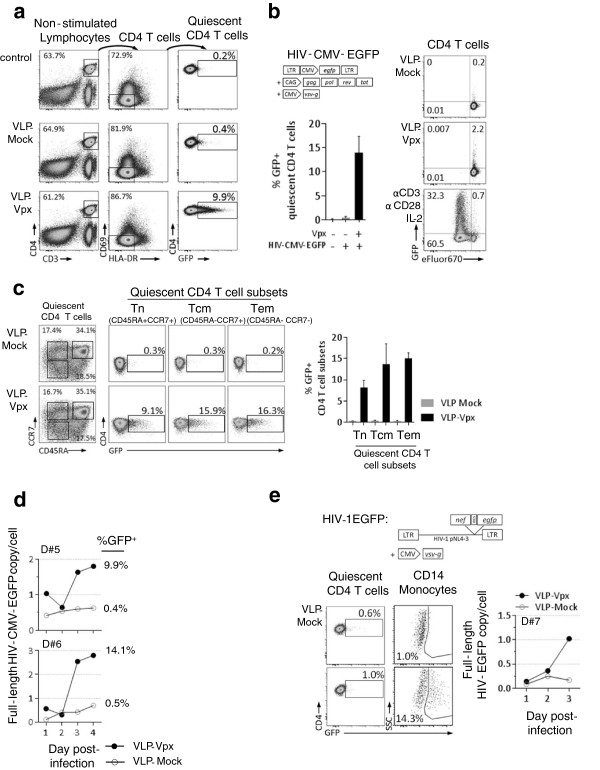
**Vpx treatment of quiescent CD4**^**+**^**T-cells results in increased susceptibility to HIV-1 infection.a**. Impact of Vpx treatment on quiescent CD4^+^ T-cells susceptibility to HIV-CMV-EGFP infection. Upper right panel: expression vectors used to produce HIV-CMV-EGFP virions. Left panel: PBMCs were treated 12 h with VLP-Mock or VLP-Vpx then infected with HIV-CMV-EGFP or uninfected (control). Proportions of EGFP positive quiescent CD4^+^ T-cells (CD69- HLA-DR-) were assessed 4 days post infection. Lower right panel: Results are expressed as percentage of EGFP positive cells. Graphic shows mean and standard deviation for 6 healthy donors. **b**. PBMCs were cultured as in **a**. after staining with the eFluor670 proliferation dye. Cell division and EGFP expression were assessed in CD4^+^ T-cells (a representative experiment is shown). **c**. Impact of Vpx on quiescent CD4^+^ T-cell subsets susceptibility to HIV-CMV-EGFP infection. Left panel: gating strategy to define CD4^+^ T-cell subsets: Naïve (Tn; CD45RA^+^, CCR7^+^), Central Memory (Tcm; CD45RA^-^, CCR7^+^) and Effector Memory (Tem; CD45RA^-^, CCR7^-^). Middle panel: Permissiveness of Tn, Tcm and Tem to HIV-CMV-EGFP performed as described in **a** (a representative experiment; n = 3). **d**. Kinetics of full length HIV-CMV-EGFP DNA accumulation quantified by quantitative PCR in purified non-stimulated CD4^+^ T-cells from 2 healthy donors #5 and #6. Proportions of EGFP positive quiescent CD4^+^ T-cells (CD69- HLA-DR-) assessed by flow cytometry 4 days post infection by HIV-CMV-EGFP are indicated on the right. **e**. Effect of Vpx on the permissiveness of quiescent CD4^+^ T-cells to infection by HIV-EGFP. Upper panel: expression vectors used to produce HIV-EGFP virions. Lower left panel: PBMCs were cultured as in **a**. except cells were infected with HIV-EGFP. Proportions of EGFP positive quiescent CD4^+^ T-cells (CD69- HLA-DR-) and CD14^+^ monocytes were assessed 4 days post infection for 3 donors. Lower right panel: Full length HIV-EGFP DNA accumulation was performed as in **d**. for 1 donor.

To test this hypothesis, we exposed unstimulated peripheral blood mononuclear cells (PBMCs) to viral-like particles containing the SIV accessory protein Vpx_mac251_ (VLP-Vpx), which counteracts SAMHD1-mediated restriction by triggering its proteasomal degradation [12,13]. PBMCs treated with VLP-Vpx or empty VLPs (VLP-Mock) were analyzed for SAMHD1 expression by flow cytometry. An average of 16% of VLP-Vpx treated resting CD4^+^ T-cells were SAMHD1 negative at 96 hours post-exposure (Figure 1c), whereas most monocytes (90.2%) lost the expression of SAMHD1 after 48 hrs of VLP-Vpx treatment (Additional file 1: Figure S1). The difference in Vpx-mediated loss of SAMHD1 in quiescent CD4^+^ T-cells and monocytes might result from discrepancies in nuclear/cytoplasmic exchange [18]. Indeed, we have recently shown that Vpx-mediated degradation of SAMHD1 requires its nuclear export [19,20]. In support of this hypothesis, loss of SAMHD1 expression was observed in more than 80% of T-cell receptor (TCR)-stimulated CD4^+^ lymphocytes expressing Vpx (Figure 1d). We next evaluated the effect of SAMHD1 loss on HIV-1-resistance phenotype of resting CD4^+^ T-cells. Restriction of HIV-1 replication in quiescent T-cells has been attributed to blocks at both the reverse transcription step and viral gene expression (e.g.: lack of transcription factors such as NF-kB and CyclinT required for viral transcription) [21,22]. Thus, to bypass the transcriptional block, we performed single-round infection experiments using an HIV-1 based lentiviral vector carrying an EGFP cassette under the transcriptional control of the CMV promoter (HIV-CMV-EGFP). Unstimulated PBMCs isolated from healthy donors were exposed for 12 hrs to VLP-Vpx or VLP-Mock and subsequently infected with HIV-CMV-EGFP. As expected, no EGFP was detected when cells were treated with VLP-Mock (Figure 2a), while, VLP-Vpx treatment resulted in EGFP expression in quiescent (HLA-DR^-^, CD69^-^) CD4^+^ T-cells (Figure 2a). An average of 14% of VLP-Vpx treated resting CD4^+^ T lymphocytes expressed EGFP, while less than 1% of cells exposed to VLP-Mock were EGFP+ (Figure 2a and Additional file 1: Figure S2a). As a control, VLP-Vpx treatment enhanced the permissiveness of CD14^+^ monocytes to HIV-CMV-EGFP (Additional file 1: Figure S3b). These results indicate that Vpx could alleviate the post-entry block to HIV-1 infection of unstimulated T-cells. Importantly, VLP-Vpx treatment was not associated with CD4^+^ T-cell activation (Additional file 1: Figure S2c) or proliferation (Figure 2b). Vpx increased the susceptibility of non-cycling quiescent T-cells (eFluor^High^) (Figure 2b) while it had no effect on the permissiveness of activated dividing T-cells (eFluor^Low^) despite high level expression of SAMHD1 (Additional file 1: Figure S2d). In addition, Vpx increased HIV-1 infection of all resting CD4^+^ T subsets, including highly refractory naïve cells (Tn) (Figure 2c).

We then focused our attention on viral reverse transcription, which is initiated in most HIV-1 exposed T-cell subsets [6]. Completion of this step is, nonetheless, reached in resting CD4+ T-cells at a much slower rate [1-6]. We asked whether SAMHD1 is responsible for the efficient reverse transcription block in resting T-cells, as it has been demonstrated for myeloid cells [13,17]. PBMCs were exposed to VLP-Vpx or VLP-Mock and infected with HIV-CMV-EGFP. The kinetics of reverse transcription leading to production of full-length HIV-1 DNA in unstimulated CD4+ T-cells was determined by quantitative PCR. We observed that both the amount and the rate of reverse transcription leading to the production of full length viral DNA were enhanced in Vpx treated resting CD4+ T-cells compared to VLP-Mock treated counterparts (Figure 2d). These results show that VLP-Vpx overcomes the restriction of HIV-CMV-EGFP infection in resting CD4^+^ T-cells by promoting the accumulation of full length reverse transcripts. We next verified whether the observed effect of Vpx applies to wild type HIV-1. For this purpose, unstimulated PBMCs were treated with VLP-Mock or with VLP-Vpx and subsequently infected with HIV-1 expressing EGFP (HIV-EGFP) (Figure 2e). Following infection, we did not detect significant EGFP expression in both VLP-Mock- and VLP-Vpx-treated resting CD4^+^ T-cells (Figure 2e and Additional file 1: Figure S3a). This is consistent with the HIV-1 LTR transcriptional block associated with their quiescent status and confirms that the analyzed CD4+ T-cells are indeed in a resting state. As a control, VLP-Vpx enhanced the permissiveness to HIV-EGFP of CD14^+^ monocyte population (Figure 2e and Additional file 1: Figure S3a). A potential infectivity defect was ruled out, since TCR-mediated activation of T-cells efficiently induced EGFP expression (Additional file 1: Figure S3b). Interestingly, while no EGFP positive cells were detected in resting CD4+ T-cells, an accumulation of HIV-1 full length DNA was observed in VLP-Vpx treated cells (Figure 2e). Thus, Vpx promotes the accumulation of full-length viral DNA following the infection of resting CD4+ T-cells, but does not relieve the transcriptional block required for viral gene expression. The ability of Vpx to promote infection was further confirmed in another model of resting lymphocytes (Additional file 1: Figure S4). Purified CD4+ T cells were activated with PHA and cultured in IL-2 for 14-20 days, until disappearance of the CD69 and Ki67 activation markers [23]. Treatment of such cells with VLP-Vpx induced SAMHD1 loss in a large fraction of the cells, and a 6-fold increase in their sensitivity to HIV-CMV-GFP infection (Additional file 1: Figure S4). Importantly, the majority of infected EGFP-positive cells were found in the SAMHD1-negative cell subset (Additional file 1: Figure S4). Taken together, these results indicate that Vpx, acting through SAMHD1, facilitates infection of resting CD4+ T-cells by promoting the accumulation of fully reverse transcribed viral DNA in quiescent lymphocytes.

**Figure 3:**
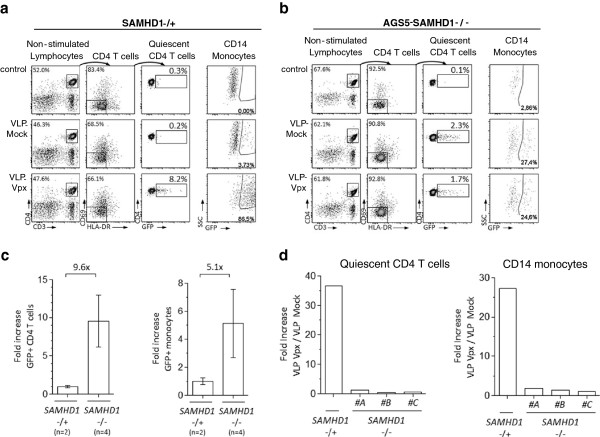
**Quiescent CD4**^**+**^**T-cells from Aicardi-Goutières Syndrome patients, with inactivating mutation of*SAMHD1*(AGS-5), display a Vpx-independent susceptibility to HIV-1 infection.a**. Impact of Vpx treatment on susceptibility to HIV-CMV-EGFP infection of quiescent CD4^+^ T-cells (CD69- HLA-DR-) and CD14^+^ monocytes from individuals with heterozygous AGS-5 *SAMHD1*^-/+^ mutation (n = 2). PBMCs were treated 12 h with VLP-Mock or VLP-Vpx then infected with HIV-CMV-EGFP or left uninfected (control). Proportions of EGFP positive quiescent CD4^+^ T-cells were assessed 4 days post infection. **b**. PBMCs from AGS-5 *SAMHD1*^-/-^ patients were treated and analyzed as in **a**. (n = 4). **c**. Impact of *SAMHD1* homozygous mutation on permissivity to HIV-CMV-EGFP infection of CD4^+^ T-cell (left panel) and monocytes (right panel). Results are expressed as a fold increase of GFP positive cells among quiescent CD4^+^ T-cells, comparing *SAMHD1*^-/-^ patients to *SAMHD1*^-/+^ individuals. **d**. Effect of Vpx treatment on the permissiveness of quiescent CD4^+^ T-cell (left panel) and monocytes (right panel) derived from *SAMHD1*^-/-^ patients (n = 3) and *SAMHD1*^-/+^ (n = 1) to HIV-CMV-EGFP. Results are presented as fold increase comparing the percentage of GFP positive cells in VLP-Vpx to VLP-Mock treated samples.

To confirm the role of SAMHD1 in the ability of Vpx to overcome HIV-1 restriction in quiescent CD4+ T-cells, we used PBMCs isolated from 4 Aicardi-Goutières syndrome patients harboring homozygous inactivating mutations in the *SAMHD1* gene (AGS-5, referred to as *SAMHD1*^-/-^) [24]. Heterozygous donors for these *SAMHD1* mutations (referred to as *SAMHD1*^-/+^) were used as controls. We first assessed the intrinsic susceptibility of unstimulated *SAMHD1*^-/-^ and *SAMHD1*^-/+^ CD4+ T-cells to HIV-CMV-EGFP infection. While heterozygous deletion of *SAMHD1* did not affect the intrinsic resistance of unstimulated PBMCs to HIV-CMV-EGFP infection, homozygous deletion increased the susceptibility of both quiescent CD4+ T-cells and monocytes (Figure 3a, b, c), indicating that SAMHD1 is required to mediate HIV-1 restriction in resting CD4+ T-cells. Remarkably, VLP-Vpx treatment did not further enhance permissiveness of *SAMHD1*^-/-^ resting CD4+ T-cells (Figure 3b, d). However, the restrictive phenotype of *SAMHD1*^-/+^ cells is alleviated after VLP-Vpx delivery (Figure 3a, d), indicating that SAMHD1 is required for Vpx to overcome HIV-1 restriction in T-cells. Overall, these results demonstrate that SAMHD1 acts as an effective HIV-1 restriction factor in non-cycling resting CD4^+^ lymphocytes.

## Conclusion

The demonstration that SAMHD1 restricts HIV-1 replication in quiescent CD4+ T-cells could have an important implication in our understanding of HIV-1-mediated CD4+ T-cell depletion and establishment of the viral reservoir, two of the HIV/AIDS hallmarks. It has recently been shown that abortive HIV-1 reverse transcription in resting CD4+ T-cells leads to the accumulation of cytoplasmic viral nucleic acids that trigger a host defense program eliciting a coordinated proapoptotic and proinflammatory response [25,26]. By preventing completion of reverse transcription in quiescent CD4^+^ T-cells, SAMHD1 could contribute to their depletion. The HIV-1 reservoir, which mainly consists of quiescent CD4^+^ T-cells that harbor integrated silent provirus, represents a major barrier to viral eradication by antiretroviral therapy. It has recently been shown that chemokines can facilitate early steps of HIV-1 replication in resting CD4^+^ T-cells, leading to latency [27]. It will be of importance to determine whether chemokines regulate SAMHD1 activity and facilitate the generation of latently infected cells in vivo. The regulation of SAMHD1 activity remains an important area of study. In this regard, we observed that SAMHD1 restriction activity does not correlate with its expression levels. Indeed, although SAMHD1 expression is independent of the activation state of CD4^+^ T-cell, its restriction activity is witnessed only when the cells are in a quiescent state (Additional file 1: Figure S2a and S2d). SAMHD1 activity could be regulated through post-translational modifications and/or through the expression of a cellular partner in non-cycling cells, including quiescent CD4^+^ T-cells. Additionally, SAMHD1 activity can also be regulated through the expression of splice variants lacking the enzymatic activity [28]. Given that dN supply only partially rescues HIV-1 reverse transcription in resting CD4+ T-cells, one can ask whether the restriction imposed by SAMHD1 is fully or partially due to its dNTP triphosphohydrolase activity. Interestingly, it has recently been shown that SAMHD1 is a nucleic acid binding protein that displays a preference for RNA over DNA [29]. Further studies are required to elucidate the mechanism by which SAMHD1 restricts HIV-1 in resting CD4+ T-cells. Deciphering the functional interaction between HIV-1 and SAMHD1 will lead to a better understanding of the damage imposed by this virus to the immune system and the progression towards AIDS.

## Methods

### Cell extract and western blot analysis

CD14^+^ and CD4^+^ T-cells were purified using CD14^+^ microbeads or CD4^+^ T-cell isolation kit (Miltenyi Biotec). CD14^+^ monocytes were treated 2 hours with VLP-Vpx before preparation of whole cell extracts (WCE). WCE were prepared with buffer containing 0.5% Triton X-100, 150 mM NaCl, 10 mM KCL, 1.5 mM MgCl2, 0.5 mM EDTA 10 mM β-mercaptoethanol, 0.5 mM PMSF. Cell lysates were boiled in SDS sample buffer and resolved on a SDS-PAGE gel (Biorad). Proteins were liquid-transferred (Biorad) to nitrocellulose membrane in transfer buffer (20% methanol, 25 mM Tris, 192 mM Glycine, 0.037% SDS) during 90 min at 100 V. Western blotting was performed using the following antibodies: mouse anti-SAMHD1 (Abcam #AB67821) and rabbit anti-Erk1/2 (Cell Signaling Technology).

### Immunofluorescence

Purified CD4^+^ T-cells were stimulated for 3 days with anti-CD3, anti-CD28 and IL-2 and transduced with Vpx expressing retroviral construct [13]. Two days post transduction, cells were harvested and fixed in PBS with 4% paraformaldehyde and 2% sucrose, and permeabilized with 0.5% Triton X-100, 20 mM Tris (pH 7.6), 50 mM Nacl, 3 mM MgCl2, and 300 mM sucrose. Wash and antibody incubation steps were performed in PBS-0.1%Tween. Cells were stained with Anti-SAMHD1 (Abcam #AB67821) and Vpx was stained with anti-HA (Covance). Secondary antibodies were purchased from Invitrogen. Nuclei were stained with DAPI in mounting media (Vectashield; Vector Labs) and images were collected on a Zeiss Axioimager Apotome.

### Plasmids

SIV3^+^ was kindly provided by N. Manel. SIV- was a gift from J. Luban. HA-Vpx_mac251_ was subcloned in pOz-IL2Rα expression vector. pHRET was kindly provided by C. Mettling. pBR-NL4-3-IRES-EGFP was a gift of F. Kirchhoff. psPAX2 packaging plasmid was obtained from Addgene (D. Trono). MMLV packaging plasmid, A-MLV envelope and pMD2-G VSV-G envelope were previously described [13].

### Virus-Like Particles (VLP) and virus production

VLPs and viral particles were produced from 293 T-cells using the standard phosphate calcium transfection protocol. For VLPs, 293 T-cells were transfected with 8 μg SIV3^+^and 2 μg pMD2-G VSV-G encoding plasmid (VLP-Vpx) or with 8 μg SIV- and 2 μg pMD2-G (VLP-Mock). Media were replaced 16 h after transfection, and VLPs were harvested 48 h later, filtered at 0.45 μm and concentrated 100 times by ultracentrifugation. For virus production, HIV-EGFP was produced by transfection of 10 μg of pBR-NL4-3-IRES-EGFP and 1 μg of pMD2-G. HIV-CMV-EGFP was produced by transfection of 5 μg of pHRET, 5 μg of psPAX2 packaging vector, and 2.5 μg of pMD2-G. For MLV transduction particles, 293 T-cells were transfected with 5 μg pOz HA-Vpx_mac251_ construct, 2.5 μg MMLV packaging plasmid, and 2.5 μg A-MLV envelope encoding plasmid. When required, p24 concentration was measured by ELISA (Innogenetics).

### Infection

PBMCs were isolated from blood samples by Ficoll gradient (Eurobio), cultured 12 h in presence of VLP- Vpx_mac251_ or VLP-Mock at a density of 2 million cells per well (24 well plates) in 300 μl of 10% FCS supplemented complete RPMI (R10) (Invitrogen). Infection was then performed by addition of 300 μl of R10 diluted HIV-CMV-EGFP (1 μg) or HIV-EGFP (800 ng) for 4 days before analysis. As control, TCR stimulated PBMCs were cultured for 3 days on plate-bound anti-CD3 antibody (5 μg/ml) (Miltenyi Biotec) in presence of 1 μg/ml anti-CD28 antibody and IL-2 (20U/ml) (Roche) and infected with HIV-CMV-EGFP (1 μg) or HIV-EGFP (800 ng) for 2 days before analysis. Optimal viral inoculums were determined by titration using TCR stimulated PBMCs. Alternatively, after thawing, Trypan Blue viability assessment and TURBO^TM^ DNase (Ambion) treatment at 37°C for 1 hour were performed, and cryopreserved PBMCs were cultured in aforementioned conditions.

### Flow cytometry

Non-cycling, quiescent CD4^+^ T-cells and monocytes were analyzed using the following antibodies and dye: Brilliant-Violet421 anti-CD3 (UCHT1), PeCy7 anti-CD4 (RPA-T4), PE anti-CD69 (FN50), APC anti-HLA-DR (TU36), V500 anti-CD14 (M5ED), PeCy7 anti-CCR7 (3D12), PE anti-CD45RA (HI100) (BD Biosciences), PerCP anti-CD45 (5B1) (Miltenyi Biotec), Cell-Proliferation Dye eFluor® 670 (eBiosciences) and Alexa-488 Anti-SAMHD1 (I-1918) (O. Schwartz). Cells were analyzed on MACSQuant Analyzer (Milteny Biotec). Data analyses were performed on FlowJo (TreeStar inc).

### Quantification of viral full-length DNA

Prior infection, viral stocks were treated for 1 h at 37°C with 100U/ml of TURBO^TM^ DNase (Ambion). PBMCs (2 X 10^6^ cells) were infected with 1000 ng of HIV-CMV-EGFP or with heat inactivated HIV-CMV-EGFP. CD4^+^ T-cells were purified from cultured PBMCs by depleting non CD4^+^ T-cell, using CD4^+^ T-cell isolation kit (Miltenyi Biotec) (Average purification yield of 97%). Genomic DNA was extracted using the QIAmp DNA blood minikit (Qiagen). Full-length viral DNA quantification was performed by quantitative PCR using primers annealing in the 5’LTR-U5 and *gag* regions. PCR measurements were performed in duplicate using SYBR Green (Qiagen). Amplifications were carried out in the LightCycler480 (Roche). The average of the technical duplicates was normalized to GAPDH levels using the comparative CT method (2ΔΔCT).

### Preparation of resting post-activated CD4+ T cells and lentiviral vector transduction

CD4+ T cells were isolated by positive selection as described above (Miltenyi Biotec). Resting CD4+ T cells were activated with 1ug/ml phytohemagglutinin (PHA) and 100 U/ml Interleukin 2 (IL-2) and cultured in fresh medium containing IL-2 for 14 to 20 days [23]. The activation state was monitored every few days by flow cytometry after staining with PE-coupled CD69 and FITC-coupled Ki67 (BD Pharmingen). Resting post-activated cells were treated with Vpx-VLPs or Mock-VLPs for 3 hours, washed and incubated overnight with HIV-CMV-EGFP. The following day, cells were washed and incubated in fresh medium containing IL-2 for 96 hours. Percentages of EGFP positive and SAMHD1 negative cells were then assessed by flow cytometry.

## Competing interests

The authors declare that they have no competing interests.

## Authors’ contributions

BD performed most of the experiments. AC and CCB, performed some experiments. OS and DA designed and performed the experiment shown in Additional file 1: figure S4. GR and YC provided AGS5 cells. BD and MB designed the experiments and wrote the manuscript. All authors read and approved the final manuscript.

## Supplementary Material

Additional file 1 SAMHD1 restricts HIV-1 replication in quiescent CD4+ T-cells. Click here for file
