# Supplementary material for: SAMHD1 restricts HIV-1 reverse transcription in quiescent CD4+ T-cells
Source: Retrovirology. 2012 Oct 23;9:87. doi: 10.1186/1742-4690-9-87 (PMC3494655; doi:10.1186/1742-4690-9-87)
Supplement: Additional file 1 — SAMHD1 restricts HIV-1 replication in quiescent CD4+ T-cells. [file 1742-4690-9-87-S1.pdf]

# SAMHD1 restricts HIV-1 replication in quiescent $CD4^+$ T-cells.

Benjamin Descours, Alexandra Cribier, Christine Chable-Bessia, Diana Ayinde, Gillian Rice, Yanick Crow, Ahmad Yatim, Olivier Schwartz, Nadine Laguette and Monsef Benkirane.

**Supplementary figures and text.**

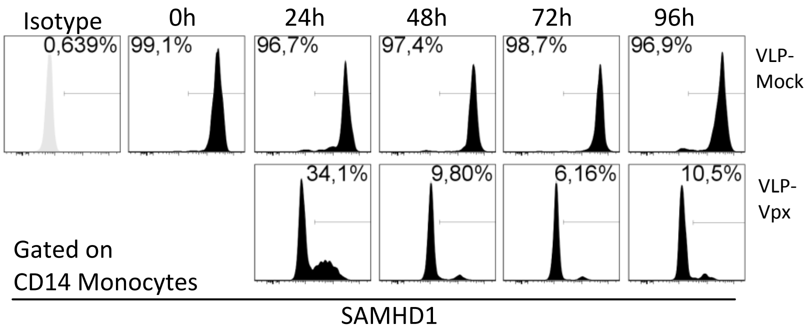


**Supplementary Figure 1. Loss of SAMHD1 expression in monocytes after Vpx treatment.** Kinetic of SAMHD1 loss in CD14 monocytes after VLP-Vpx treatment was assessed by Facs using anti-SAMHD1 antibody (Representative experiment, n=2).

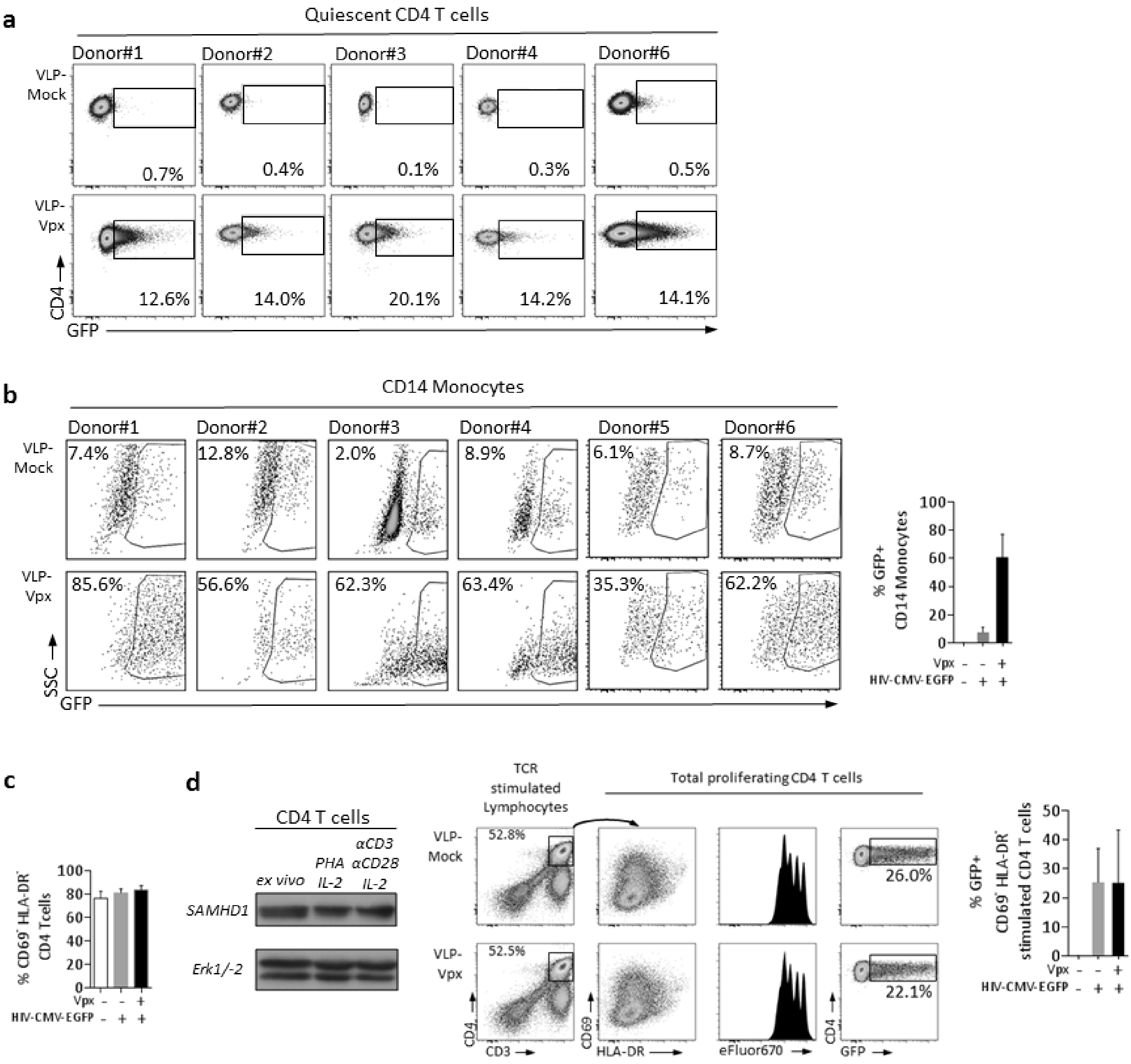


**Supplementary Figure 2. Quiescent CD4+ T-cells and CD14+ monocytes display increased susceptibility to HIV-1 infection after Vpx treatment. a.** Dot plots illustrating the susceptibility of quiescent CD4+ T-cells after VLP-Mock or VLP-Vpx treatment followed by HIV-CMV-EGFP infection as represented in Figure 2C, in healthy donors 1 to 4 and 6 (healthy donor #5 is shown in Figure 2a). **b**. Left panel: As in **a.**, dot plots illustrating the susceptibility of CD14+ monocytes VLP-Mock or VLP-Vpx treatment followed by HIV-CMV-

EGFP infection in healthy donors 1 to 6. Right panel: Results are expressed as percentage of GFP positive cells. Graphic shows mean and standard deviation for these 6 healthy donors. **c**. Proportions of resting (CD69- HLA-DR-) CD4 T cells were assessed in 6 donors in uninfected, VLP-Mock and VLP-Vpx treated cells. Resultes are expressed as percentage of total CD4 T cells. **d.** Impact of Vpx treatment on TCR stimulated CD4 T cell susceptibility to HIV-CMV-EGFP infection. Left panel: after staining with the eFluor670 proliferation dye, PBMCs were stimulated for 3 days with anti-CD3 and anti-CD28 antibodies and IL-2, treated with VLP-Mock or VLP-Vpx for 12 hours and infected by HIV-CMV-EGFP for 2 days before FACS analysis (Representative dot plots from one experiment out of three is shown). Right panel: Results are expressed as percentage of GFP positive cells. Graphic shows mean and standard deviation for 3 healthy donors.

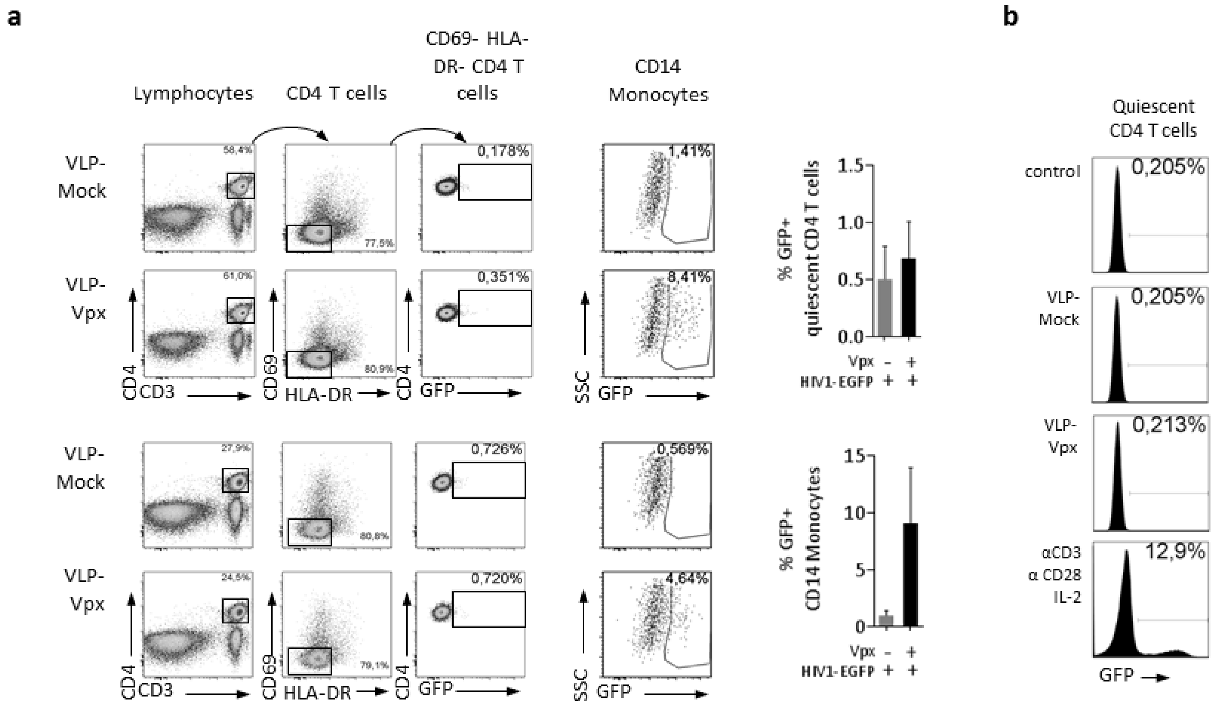


**Supplementary Figure 3. Effect of Vpx on the permissiveness of quiescent CD4$^{+}$ T-cells to HIV-EGFP infection. a.** Left panel: Dot plots illustrating the susceptibility of quiescent CD4$^{+}$ T-cells and CD14$^{+}$ monocytes after VLP-Mock or VLP-Vpx treatment followed by HIV-EGFP infection as represented in **Figure 2a**, in healthy donors #5 and #6 (healthy donor #7 is shown in Figure 2e). Right panel: results are expressed as percentage of GFP positive cells. Graphic shows mean and standard deviation for 3 healthy donors. **b.** Flow cytometry histograms showing HIV-EGFP infection susceptibility of non-stimulated CD4 T cells treated with VLP-Mock or VLP-Vpx and of CD4 T cells stimulated through the TCR as describe in supplementary **figure 2d**.

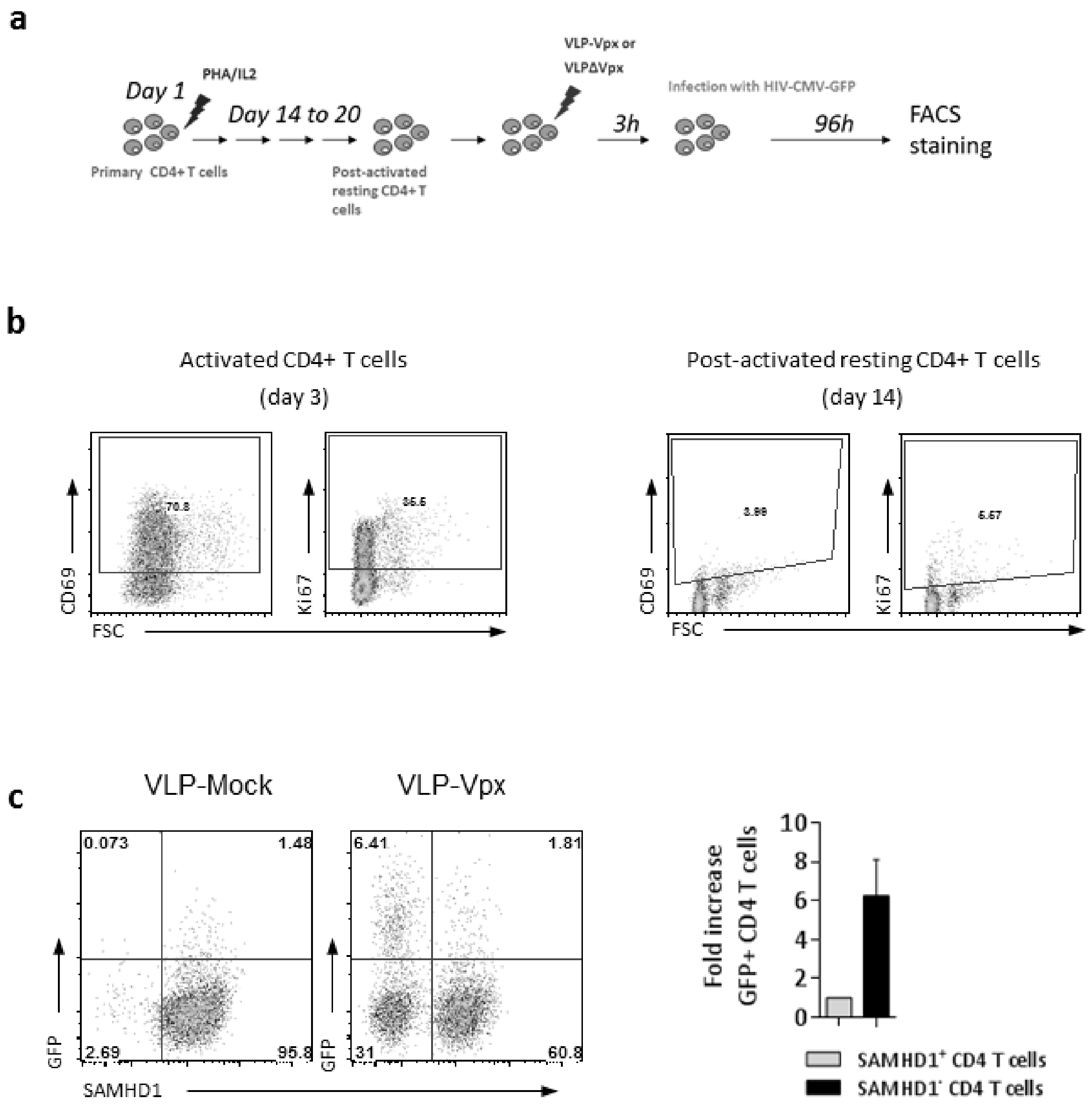


**Supplementary Figure 4.** Enhanced permissiveness of purified CD4+ T-cells by VLP-Vpx correlates with loss of SAMHD1 expression. **a.** Experimental outline. CD4+ T cells were purified from PBMCs and activated with phytohemagglutinin (PHA) and IL-2. Cells were maintained in IL-2 for 14 to 20 days, until disappearance of the activation markers CD69 and Ki67. The resulting “resting post-activated cells” were then treated for 3h with VLP-Mock or

VLP-Vpx and infected with HIV-CMV-EGFP lentiviral vector. Cells were then analyzed for GFP and SAMHD1 expression by flow cytometry at 96h post-infection. **b.** Left panel: Analysis of expression of CD69 and Ki67 activation markers by flow cytometry in resting post activated cells compared to activated cells (at day 3 post PHA stimulation, right panel). **c.** Vpx enhances lentiviral vector infection of post-activated resting CD4+ T cells. One representative donor out of seven is shown (right panel). The graph (right panel) represents mean ± SEM of the fold increase in GFP expression in SAMHD1 positive and negative populations following incubation with VLP-Vpx.
